# Phytochemical screening, antioxidant, and antimicrobial activities of the crude leaves’ extract from *Ipomoea batatas* (L.) Lam

**DOI:** 10.4103/0973-1296.80682

**Published:** 2011

**Authors:** Márcia Thaís Pochapski, Eliana Cristina Fosquiera, Luís Antônio Esmerino, Elizabete Brasil dos Santos, Paulo Vitor Farago, Fábio André Santos, Francisco Carlos Groppo

**Affiliations:** *Department of Pharmacology, Anesthesiology and Therapeutics, Piracicaba Dental School, University of Campinas, Piracicaba, SP, Brazil*; 1 *Department of Dentistry, Ponta Grossa State University, Ponta Grossa, Paraná, Brazil*; 2 *Department of Phamarcy, Ponta Grossa State University, Ponta Grossa, Paraná, Brazil*

**Keywords:** Antimicrobial activity, antioxidant activity, convolvulaceae, phenolic content, sweet potato

## Abstract

**Background:**

*Ipomoea batatas* (L.) Lam., popularly known as sweet potato (SP), has played an important role as an energy and a phytochemical source in human nutrition and animal feeding. Ethnopharmacological data show that SP leaves have been effectively used in herbal medicine to treat inflammatory and/or infectious oral diseases in Brazil. The aim of this research was to evaluate the phytochemical, antioxidant, and antimicrobial activities of the crude leaves’ extract of SP leaves.

**Materials and Methods:**

The screening was performed for triterpenes/steroids, alkaloids, anthraquinones, coumarins, flavonoids, saponins, tannins, and phenolic acids. The color intensity or the precipitate formation was used as analytical responses to these tests. The total antioxidant capacity was evaluated by the phosphomolybdenum complex method. Antimicrobial activity was made by agar disk and agar well diffusion tests.

**Results:**

The phytochemical screening showed positive results for triterpenes/steroids, alkaloids, anthraquinones, coumarins, flavonoids, saponins, tannins, and phenolic acids. Total contents of 345.65, 328.44, and 662.02 mg were respectively obtained for alkaloids, anthraquinones, and phenolic compounds in 100 g of the dry sample. The total antioxidant capacity was 42.94% as compared to ascorbic acid. For antimicrobial studies, no concentration of the SP freeze dried extract was able to inhibit the growth of *Streptococcus mutans, S. mitis, Staphylococcus aureus*, and *Candida albicans* in both agar disk and agar well diffusion tests.

**Conclusions:**

SP leaves demonstrated the presence of secondary metabolites with potential biological activities. No antimicrobial activity was observed.

## INTRODUCTION

*Ipomoea batatas* (L.) Lam., popularly known as the sweet potato (SP), has played an important role as an energy and a phytochemical source in human nutrition and animal feeding. SP continues to be of remarkable economic value as the sixth most plentiful food crop in the world.[1–4] This tuberous root is a high-quality source of carbohydrates, dietary fiber, vitamin A (as β-carotene), vitamin B_6_, vitamin C, manganese, copper, potassium, and iron.[2] Recently, studies on SP have focused on its antioxidant capacities due to the increased content of phenols, flavonoids, β-carotene, anthocyanins, and caffeoylquinic acid derivatives.[2 5–8] Other reports have reported its medicinal use, specifically its antidiabetic and antiviral properties.[9,10]

Despite the remarkable use of its tuberous roots, there is a lack of detailed data related to the phytochemical composition and biological properties for SP leaves.[11] A few studies have performed quantitative analyses for some nutrients and antinutrients from SP leaves. The antinutrient composition was determined by its phytic acid, cyanide, tannins, and total oxalate values.[12] An antimutagenic potential was regarded as isolated caffeoylquinic acid derivatives from SP leaves.[7,13] A hidroalcolic extract from SP leaves demonstrated no anti-inflammatory effect by the rat paw edema induced by compound 48/80.[14] Moreover, ethnopharmacological data have shown that the leaves from SP have been used successfully as a herbal medicine for inflammatory and/or infectious oral diseases in Brazil.[3]

The aim of this research was to perform phytochemical, antioxidant and antimicrobial activities on the crude extract of SP leaves.

## MATERIALS AND METHODS

### Plant material

The plant material was collected from the region of Ponta Grossa, in the state of Paraná, Southern Brazil, from September to November 2009, and identified by a specialist. The voucher material was deposited at Ponta Grossa State University Herbarium (HUPG). The collection number was HUPG #17.033.

### Preparation of the crude extract of SP leaves

A hydroethanolic extract was prepared from SP leaves as a dried powder with a mean size of particle diameter of 500 μm and humidity content of 7.12% (w/w). Twenty grams of powder was mixed into 100 mL of ethanol 70% (V/V) and submitted to dynamic maceration for 7 days at room temperature. Then, the extract was filtered with a qualitative cellulose filter paper (particle retention: 20-25 μm). After filtration, the SP crude extract was concentrated under reduced pressure of 300-500 mmHg at 50-60°C. Subsequently, the extract was stored in the deep freezer at -80°C, and later dried for 2 days in a freeze dryer at -40°C and 400 μHg vacuum.

### Phytochemical screening

The filtrated SP crude extract (200 mL) was concentrated under reduced pressure and partitioned by sequential extractions with *n*-hexane, chloroform, ethyl acetate, and ethanol 70% (V/V). These four fractions were evaluated by phytochemical qualitative reactions for usual plant secondary metabolites. The screening was performed for triterpenes/steroids, alkaloids, anthraquinones, coumarins, flavonoids, saponins, tannins, and phenolic acids.[15–20] The color intensity or the precipitate formation was used as analytical responses to these tests.

### Determination of the total alkaloid content

Regarding the previous data from phytochemical screening, the alkaloid content was achieved in triplicate by liquid-liquid extraction and gravimetric analysis. Briefly, the chloroform fraction (20 mL) was successively partitioned with HCl, 1, 0.5, and 0.25 mol/L. The aqueous phase was collected, alkalinized with NH_4_OH, 6 mol/L, and partitioned with chloroform. This organic phase was then evaporated to dryness on a water bath (60°C) in a previously weighed porcelain capsule. For the hydroethanolic fraction (20 mL), alcalinization with NH_4_OH, 6 mol/L, partition with chloroform, and evaporation to dryness were also carried out. The mass of the residual solid from both chloroform and hydroethanolic fractions was accurately determined. The total alkaloid content from free base and salt forms was expressed as milligrams of alkaloids per 100 g of the dry sample.

### Determination of the anthraquinone content

Also concerning phytochemical screening, the anthraquinone content was spectrophotometrically quantified in triplicate, after a suitable pretreatment. A volume of 0.5 mL of ethyl acetate fraction was alkalinized with 50 mg of NaHCO_3_ and oxidized with an aqueous solution of 10.5% FeCl_3_ (20 mL). The mixture was boiled under reflux for 5 min. Then, 1 mL of concentrated HCl was added and the reaction medium was kept under the same condition for 20 more minutes. At room temperature, the mixture was partitioned with diethyl ether three times. The ether phase was transferred into a 100-mL volumetric flask and the final volume was completed with this organic solvent to obtain the stock solution. Then, 10 mL of the stock solution was evaporated to dryness on a water bath (60°C). The residual solid was dissolved in 10 mL of 0.5% magnesium acetate as methanol solution. The absorbance was analyzed at 515 nm with a UV-Vis spectrophotometer. The same analytical procedure was performed for the hydroethanolic fraction. Standard solutions of 1,8-dihydroxyanthraquinone (0.005-0.06 mg/mL) in ether were similarly evaporated and treated with 0.5% magnesium acetate in methanol to achieve the analytical curve. The methanol solution was used as a control. Results were expressed as milligrams of hydroxyanthracene derivatives per 100 g of the dry sample.[21]

### Determination of the total phenolic content

The extraction of phenolic compounds was based on a modified method by Hsu *et al*.[22] Five grams of SP leaves as a dried powder was mixed with 80 mL of methanol and kept overnight. The suspension was filtered through a qualitative cellulose filter paper (particle retention: 20-25 μm) and the filtrate was diluted to 100 mL with methanol. Sample solutions (*n* = 3) were stored at 4°C in amber bottles and served as the stock solution (50 mg/mL) for subsequent analyses.

For total phenolic content determination, 200 μL of each sample was mixed with 1.4 mL purified water and 100 L of Folin-Ciocalteu reagent. After at least 30 s (but not exceeding 8 min), 300 μL of 20% Na_2_CO_3_ aqueous solution was added and the mixture was allowed to stand for 2 h. The absorbance was measured at 765 nm with a UV-Vis spectrophotometer. Standard solutions of gallic acid (10-100 ppm) were similarly treated to plot the analytical curve. The control solution contained 200 μL of methanol and suitable reagents, and it was prepared and incubated under the same conditions as the rest of the samples. Results were expressed as milligrams of gallic acid equivalent (GAE) per 100 g of the dry sample.[8,23]

### Evaluation of the total antioxidant capacity

The total antioxidant capacity was evaluated by the phosphomolybdenum complex method in triplicate. A 0.005 g mass of the SP freeze dried extract was added to 25 mL of ethanol 70% (V/V) and homogenized by vortex agitation. An aliquot of 300 μL of this hydroethanolic extract standardized to 200 μg/mL was combined in an amber vial with 3 mL of the reagent solution (0.6 mol/L sulfuric acid, 28 mmol/L sodium phosphate, and 4 mmol/L ammonium molybdate). These vials were capped and incubated in a thermal block at 95°C for 90 min. Then, the sample was cooled to room temperature and its absorbance was measured at 695 nm against a blank. A typical blank solution contained 1 mL of reagent solution and appropriate volume of the ethanol 70% (V/V), and it was incubated under the same conditions as the rest of the samples. In order to prepare ascorbic acid (200 μg/mL), 0.02 g mass was accurately weighted and completed to purified water, 100 mL. A volume of 300 μL was used as 100% of the antioxidant activity as calculated by Equation 1, where *A* represents each absorbance value.[24,25]

…(1) $$\mathrm{Antioxiden}\;\mathrm{tectivity}\;\left(\%\right)\;=\left(\frac{A_{sample}\;–\;A_{blank}}{A_{ascorbicacid}\;–\;A_{blank}\;}\right)\times 100$$

### Antimicrobial activity studies

#### Agar disk diffusion test

The SP freeze dried extract was dissolved in an aqueous solution of 50% (V/V) dimethyl sulfoxide (DMSO) in order to obtain 5%, 10%, and 15% sample solutions. As a positive control, 2% chlorhexidine digluconate was prepared using the same vehicle. The DMSO solution was used as a negative control. A volume of 10 μL of each solution was placed onto individual sterile 6.35-mm filter disks and allowed to dry at room temperature. Each disk was placed on the surface of Mueller-Hinton (MH) or brain heart infusion (BHI) agar that had been previously inoculated with standardized inoculum suspensions that match the turbidity of the 0.5 McFarland standard (1.5 × 10^8^ CFU/mL) of *Streptococcus mutans* (ATCC 25175), *S. mitis* (ATCC 903), *Staphylococcus aureus* (ATCC 25923), and *Candida albicans* (ATCC 10231) by swab streaking. The diameter of the zone of growth inhibition around each disk was determined in millimeters after 24 and 48 h of incubation at 35 ± 0.5°C. Tests were performed in triplicate.[11,26]

#### Agar well diffusion test

Each bacterial inoculum (*S. mutans, S. mitis, S. aureus, and C. albicans*) was swab streaked on MH or BHI agar as previously described. Wells of 7 mm diameter were cut into agar plates and 50 μL of sample solutions and positive control were added to each well. Plates were incubated for 24 and 48 h at 35 ± 0.5°C. The zone of growth inhibition was measured in millimeters. Tests were carried out in triplicate.[11,26]

## RESULTS AND DISCUSSION

### Phytochemical study

All results of phytochemical analysis are showed in the Table 1.

**Table 1:** SP crude extract fractions, phytochemical screening results

Metabolites	Fractions			
	n-hexane	Chloroform	Ethyl acetate	Ethanol 70% (V/V)
Triterpenes/ steroids	++	+	−	−
Alkaloids	−	+++	−	++
Anthraquinones	−	−	++	++
Coumarins	−	−	++	+
Flavonoids	−	++	+++	+++
Saponins	−	−	−	++
Tannins	−	−	−	++
Phenolic acids	−	−	−	++

+++: Strong intensity reaction

++: Medium intensity reaction

+: Weak intensity reaction

−: Nondetected

In the present study, the *n*-hexane and chloroform fractions from the SP crude extract showed positive results for triterpenes and/or steroids as measured by the Liebermann-Burchard reaction, confirming the scant data yielded by previous studies of SP leaves, which indicate the presence of phytosterols in mature and immature tubers of *I. mauritiana* Jacq.[27,28]

Alkaloids were observed as orange, red-orange, or brown-orange precipitates in the performed chloroform and residual ethanol fractions by using the Dragendorff, Bertrand, and Bouchardat method. The total alkaloid content of SP leaves was measured at 345.65 ± 15.52 mg in 100 g of the dry sample (RSD = 4.4%). An indole-type alkaloid called ipomine A was found for tuberous roots of SP.[29] Tubers of *I. mauritiana* also revealed alkaloid content.[28] However, papers that studied some nutritive and antinutritive features of SP leaves made no mention of the presence of alkaloids.[12] Due to their extensively reported pharmacological and toxicological data, the alkaloid content shown in these studies must be cautiously evaluated.[30,31]

For anthraquinones, the Bornträger reaction showed a positive red color for ethyl acetate and residual ethanol fractions from the SP crude extract. The total anthraquinone content of SP leaves was determined as 328.44 ± 8.17 mg of hydroxyanthracene derivatives in 100 g of the dry sample (RSD = 2.5%). Mors *et al*.[3] previously indicated some purgative properties for roots from *I. acetosifolia* (Vahl.) Roem. & Schult. and *Ipomoea pes-caprae* (L.) R. Br. due to their anthraquinone content. However, little data have been suggested for the presence of this particular secondary metabolite in leaves of *Ipomoea* spp.

Fluorescence was detected by the UV test (365 nm) for ethyl acetate and residual ethanol fractions from the SP crude extract which indicated the presence of coumarins. Although only a small number of studies have been undertaken for the purpose of examining coumarins from *Ipomoea* spp., a previous project has successfully isolated and characterized two coumarins (umbelliferone and scopoletin) in aerial parts of *I. cairica* (L.) sweet.[32]

Flavonoids were verified for the studied chloroform, ethyl acetate, and residual ethanol fractions when treated with the Shinoda reagent, boric acid/oxalic acid solution, and zinc/hydrochloric acid reaction. The flavonoid content has been widely investigated in *Ipomoea* species. Recently, anthocyanins, catechins, flavonols, and proanthocyanidins from SP leaves were identified and quantified using high-performance liquid chromatography combined with a photodiode-array detector.[5] Flavonoids were also verified in mature and immature tubers of *I. mauritiana*.[28] Moreover, the presence of flavonoids in *Ipomoea* spp. can be useful as a chemotaxonomic approach for assessing the status of these species.

The presence of saponins was confirmed by foam-producing properties of these secondary metabolites considering an aqueous solution obtained from the residual ethanol fraction of the SP crude extract. Saponins were also identified in SP tubers as triterpene saponins.[2]

A 2.5% gelatin aqueous solution, a 1% ferric chloride aqueous solution, and a 5% ferric ammonium sulfate in HCl, 2 mol/L, yielded positive results for tannins in the residual ethanol fraction from the SP crude extract. Tannins were previously verified in SP leaves as antinutrient components.[12]

Phenolic acids as hydroxycinnamic and hydroxybenzoic derivatives were identified in the studied SP leaves by the Carrez reagent (3.6% potassium ferrocyanide and 7.2% zinc sulfate in aqueous solutions).[5,33] Due to the radical-scavenging, antimutagenic, antidiabetes, and antibacterial properties of these organic acids,[34] recent papers have been devoted to investigating these compounds in SP.[1,7,8] A higher content of phenolic acids waas found in SP leaves as compared with those of major commercial leafy vegetables.[6] These natural products were, however, not detected in SP tuberous roots.[7]

Despite the lack of information about secondary metabolites, particularly for SP leaves, extensive data are available for Convolvulaceae. The natural products reported for the members of this family can be classified as polyketides, terpenoids (mono-, sesqui-, di-, and triterpenoids), steroids, shikimides (coumarins and benzoic and cinnamic acid derivatives), flavonoids (flavonols, anthocyanins, flavones, flavanones), xanthone, alkaloids (pyrrolidines, tropanes, indolizidines, and ergolines), and other compounds.[32] Therefore, metabolites observed in SP leaves are identical to the chemicals derived from for Convolvulaceae.

### Total phenolic content

The total phenolic content of SP leaves was 662.02 ± 28.91 mg GAE in 100 g of the dry sample (RSD = 4.4%). A previous study reported that tuberous roots of five SP varieties showed phenolic content ranging from 192.7 to 1159.0 mg GAE/100 g dry sample.[8] Therefore, present study’s results confirm earlier data derived from research on SP tuberous roots. Moreover, SP leaves can be also considered a high source of phenolic substances (e.g., flavonoids, phenolic acids, tannins, and tocopherols) with a potential medicinal use as previously demonstrated for tubers.

### Evaluation of the total antioxidant capacity

A relative antioxidant activity of 42.94 ± 0.89% (RSD = 2.0%) was obtained for the hydroethanolic extract from SP leaves as compared to ascorbic acid. This value represents a moderate potential in reducing the phosphomolybdenum complex that can suggest SP as a viable alternative source of antioxidants. Previous data showed similar results for tuberous roots of SP by different analytical methods, e.g., 1,1-diphenyl-2-picrylhydrazyl (DPPH) radical and ferric thiocyanate procedures.[8]

### Antimicrobial activity studies

For agar disk diffusion and agar well diffusion tests, all microorganisms were inhibited by the 2% chlorhexidine aqueous solution. However, no concentration from the SP freeze dried extract showed zone of growth inhibition against *S. mutans, S. mitis, S. aureus*, and *C. albicans* using either method; neither was a zone of inhibition verified using the DMSO solution. Conversely, a previous study has revealed an antimicrobial activity for three different cultivars of SP leaves against *Escherichia coli* O157:H7, *Bacillus cereus* and *S. aureus* by the use of a lyophilized leaf powder and Tryptone Soya Broth medium. The main components of this antibacterial extract were polysaccharides and proteins.[11] The negative results observed in the present study can be accounted for by the differences in the phytochemical composition and the methodology of the antimicrobial tests.

In conclusion, SP leaves demonstrated the presence of triterpenes and/or steroids, alkaloids, anthraquinones, coumarins, flavonoids, saponins, tannins, and phenolic acids as secondary metabolites with potential biological activities. Analogous results of phenolic content and antioxidant activity were observed for SP leaves as compared with the SP tuberous root. No antimicrobial activity against *S. mutans, S. mitis, S. aureus* and *C. albicans* was observed for the evaluated freeze dried material from the crude hydroethanolic extract of SP leaves.
